# Efficient Development of Gait Classification Models for Five-Gaited Horses Based on Mobile Phone Sensors

**DOI:** 10.3390/ani13010183

**Published:** 2023-01-03

**Authors:** Haraldur B. Davíðsson, Torben Rees, Marta Rut Ólafsdóttir, Hafsteinn Einarsson

**Affiliations:** 1 Department of Computer Science, University of Iceland, 101 Reykjavík, Iceland; 2 Horseday ehf., 102 Reykjavík, Iceland; 3 TöltSense Ltd., Newton Abbot TQ12 5ND, UK

**Keywords:** horse, smartphone sensors, inertial measurement unit, gait classification, machine learning

## Abstract

**Simple Summary:**

This study explored the use of mobile phone sensors to accurately classify the gaits of five-gaited horses. The data were collected from horses and riders using a mobile phone in the rider’s pocket and an existing multi-sensor gait classification system. A machine learning model was then trained to classify the gaits using input from the phone’s accelerometer and gyroscope, achieving an accuracy of 94.4%. This research demonstrates that mobile phones can be used to gather data on horse gaits, reducing the cost of large-scale studies. This efficient method for acquiring labelled data will be invaluable for ongoing research into horse riding activities.

**Abstract:**

Automated gait classification has traditionally been studied using horse-mounted sensors. However, smartphone-based sensors are more accessible, but the performance of gait classification models using data from such sensors has not been widely known or accessible. In this study, we performed horse gait classification using deep learning models and data from mobile phone sensors located in the rider’s pocket. We gathered data from 17 horses and 14 riders. The data were gathered simultaneously from movement sensors in a mobile phone located in the rider’s pocket and a gait classification system based on four wearable sensors attached to the horse’s limbs. With this efficient approach to acquire labelled data, we trained a Bi-LSTM model for gait classification. The only input to the model was a 50 Hz signal from the phone’s accelerometer and gyroscope that was rotated to the horse’s frame of reference. We demonstrate that sensor data from mobile phones can be used to classify the five gaits of the Icelandic horse with up to 94.4% accuracy. The result suggests that horse riding activities can be studied at a large scale using mobile phones to gather data on gaits. While our study showed that mobile phone sensors could be effective for gait classification, there are still some limitations that need to be addressed in future research. For example, further studies could explore the effects of different riding styles or equipment on gait classification accuracy or investigate ways to minimize the influence of factors such as phone placement. By addressing these questions, we can continue to improve our understanding of horse gait and its role in horse riding activities.

## 1. Introduction

Mobile devices have become an accepted part of our everyday lives, and with the rapid pace of technological progress, their applications are constantly evolving. With sophisticated built-in motion sensors, users expect their devices to be able to perform human activity recognition. However, the devices are not only limited to the classification of human activities, since they can also be used for animal activity classification. Gait classification has been implemented in commercial smartphone apps such as Equilab (https://equilab.horse, accessed on 1 May 2022), which can recognize four gaits (walk, trot, canter, and tölt). The work of this paper was performed in collaboration with Horseday ehf., who are working on an app that can perform equine gait classification for five-gaited Icelandic horses, i.e., it can recognize flying pace in addition to the four other gaits.

Published work on equine gait classification dates back to the landmark work by Hildebrand in 1965 [1]. In that work, he described a standard for the task, using variables derived from limb movements and step placement. Since then, specially designed motion sensor systems for horses have been used to collect data for gait events and classification. Such data have been used to predict the timing of hoof contact [2,3], to monitor lameness [4], to analyse equestrian show jumping and dressage training movements [5], and to detect indications of fatigue during training [6]. Furthermore, machine learning models using data from horse-mounted sensors for gait classification have been tested [7], and the accuracy of the gait classification models on such sensor data reached 97% in a recent study by Bragança et al. [8]. Furthermore, mobile phone sensors have been shown to have a good agreement with validated specialist IMUs when both devices are attached to the horse [9].

Moreover, Bragança et al. [8] showed that a long short-term memory (LSTM) model [10] that received the raw sensor data as the input performed only slightly worse than the model using Hildebrand’s variables. Similarly, convolutional networks have been used to classify raw accelerometer data from sensors strapped to the horses [11]. Although the models reach good accuracy using inputs from horse-mounted sensors, they still require the rider to explicitly attach sensors to the horse’s limbs and body. This inconvenience leads us to the question of what accuracy can be reached using wearable human sensors carried in the rider’s pocket. Studies on three-gaited horses (walk, trot, canter) show that gait classification can be performed using their sensor recordings [12,13]. However, it is not clear to what extent this would apply to the five-gaited Icelandic horse using smartphone sensors.

In this paper, we studied the accuracy of gait classification for all five gaits of the Icelandic horse using models trained on data from mobile phones in the rider’s pocket. We specifically studied the Icelandic horse, which can perform two additional gaits, tölt and flying pace, on top of the three standard ones, walk, canter, and trot, due to a gene mutation [14]. We used the TöltSense (https://toltsense.com, accessed on 1 May 2022) system (TS) to label the training data. In previous studies, gait labelling was performed by recording the horse with a camera in a controlled environment and labelling the gait by watching the recording [8] or by timing gait switches using a stopwatch [13]. The TS automates the gait labelling process, which makes data acquisition significantly more accessible. The system further makes the labelling process objective and feasible in a diverse and natural environment.

Our study aims to answer the following research question: What accuracy of gait classification for all five gaits of the Icelandic horse can be reached using models trained on data from mobile phones in the rider’s pocket? Our two objectives were to evaluate the accuracy of gait classification using the TS system and to evaluate the gait classification accuracy of models trained using the rotated sensor signals from mobile phones and TS gait labels. Our hypothesis was that models trained using TS labels and the rotated sensor signals from mobile phones will perform well in gait classification of Icelandic horses.

## 2. Materials and Methods

**Figure 1:**
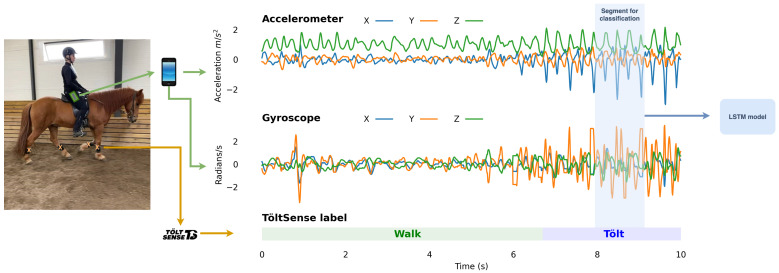
Data labelling with the TS application. The left panel shows the location of the sensors. The TöltSense sensors are strapped to the lateral aspect of the metacarpal/metatarsal bone, and the phone is placed in the rider’s pocket. The right panel shows the data from the mobile sensor after rotation to a given frame of reference (world-frame) and the gait labels as a horse switches from walk to tölt. The X and Y curves correspond to variations in the acceleration and gyroscope signal in the horizontal plane, and the Z curve corresponds to variations on the vertical axis. The sign of the signal on a given axis corresponds to the direction of the signal on that axis. The highlighted segment shows an example of the input we used for the Bi-LSTM model.

For both the mobile phone sensor model and TöltSense validation studies, the local Ethics Committee (The Icelandic Food and Veterinary Authority and the Ethics Review Board at the Royal College of Veterinary Surgeons) waived the need for a formal review and approval. It was concluded that the study is outwith the European Directive 2010/63/EU as it does not meet the threshold for causing any pain, distress, suffering or lasting harm. All the methods in each individual study were carried out in accordance with the approved guidelines and regulations. Informed consent was obtained from the owner of the animals and riders in a written manner when needed. Informed consent for publication was obtained from the rider in Figure 1.

### 2.1. The TöltSense System

To acquire labels for our training set, we used the TöltSense system (TS). The TS is a training tool designed to classify and analyse the quality of Icelandic horse gaits and provide feedback to the rider in real-time. The system is composed of motion sensors and a mobile app to report analysis results to the user. The four wireless motion sensors were attached to the horse’s lower limbs, and they were kept synchronized to within 8ms of each other. The cross-platform mobile app processes the signals and generates gait labels (see Figure 1) with up to 99.7% accuracy (see the Results Section). The TS is not based on machine learning, but on the definitions of the gaits [15] by the International Federation of Icelandic Horse Associations (FEIF). The TS is based on the principle that a handcrafted algorithm can determine gaits if the hoof-on and hoof-off timings are measured with sufficient accuracy.

### 2.2. Dataset and Labelling—TöltSense Validation

Eight Icelandic horses of varying levels of training and ability were ridden and filmed while wearing the TS equipment at a horse farm in the U.K. Some of the sessions were captured during warm-up for an oval track competition in an indoor arena. The rest were captured during a training day on an oval track.

**Figure 2:**
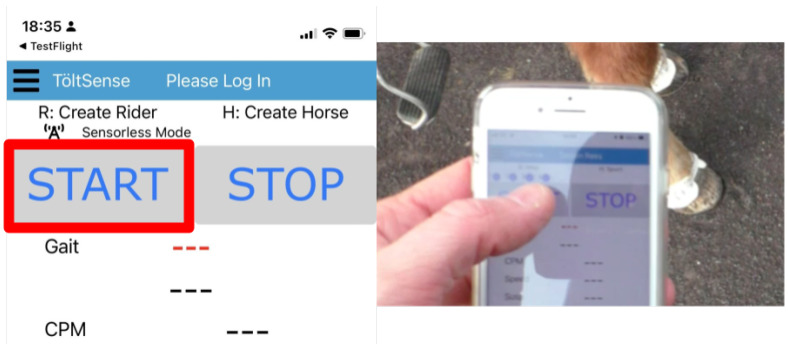
Töltsense interface and an example frame from a video of how the starting event was filmed with the press of the START button.

At the beginning of each session, the press of the TS app’s “START” button was recorded (see Figure 2). This button press initiated the creation of a log file of gait classifications and timestamps, and recording it provided a reference point to line up the video with the TS log. Each video was trimmed to start exactly when the “START” button was pressed so that the times in the video would correspond to the times in the app log.

**Figure 3:**
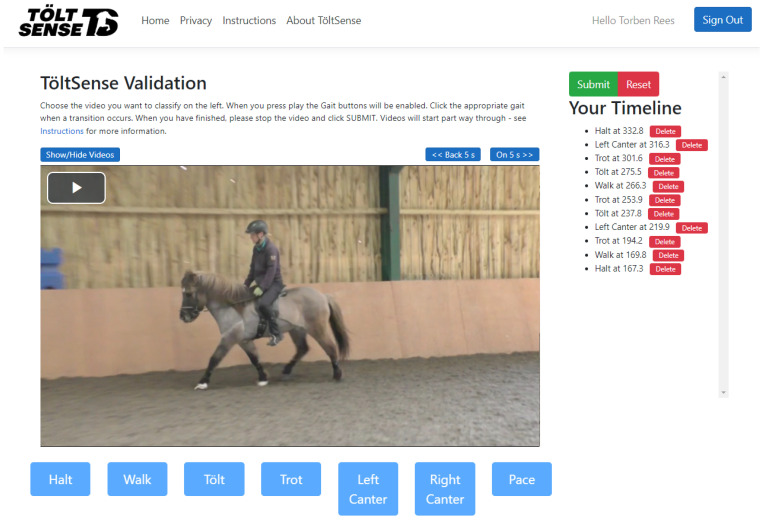
The web application used by the judges to assign gait labels to video segments.

A panel of 4 qualified Icelandic sport judges independently suggested gait labels while watching the videos, using a custom-made web application (see Figure 3). For each video, a continuous observation window of 4–5 min was defined so as to include as many transitions and gaits as possible and to avoid judges having to watch unnecessary footage. At no point were the classifications of the TS revealed to the judges.

**Figure 4:**
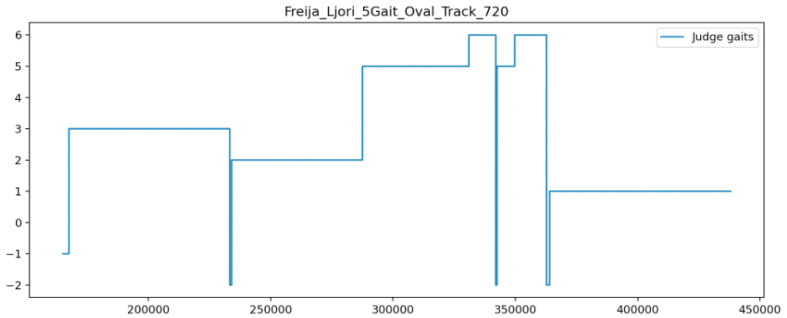
Plot of aggregate judge classifications against time, showing initial unclassified period (−1) and three periods of dispute (−2). The *y*-axis corresponds to the chosen label as defined in Table 1.

**Figure 5:**
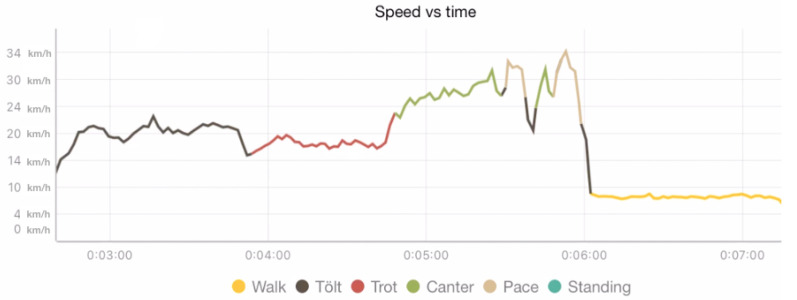
Plot of speed vs. time colour coded by gait from the TöltSense session overview screen.

A gait was determined every 250 ms by choosing the most-common label suggested during the given time interval. Such a majority vote was applied throughout the observation window, and a new data point was created whenever the majority gait changed. This processing step resulted in a series that defined the gait at any given moment during the observation window. We refer to this time series as aggregate judge classifications. An illustration of this time series is shown for a single example in Figure 4, and the speed of one horse with TS gait labels is shown in Figure 5. The values in the time series correspond to the gaits according to Table 1.

The category “No majority/disputed” was required because there were occasions where the judges did not agree on the gait. Mostly, these were brief periods around gait transitions. However, Icelandic horses often display movements that are “between gaits” (e.g., pacey tölt, or 4-beat trot), and in these cases, it is not clear which gait is being shown. If qualified observers do not agree on the gait, there is no ground truth to compare the TS against, so periods labelled as −2 were excluded from the assessment. “Not classified” appears once at the very start of each session before any classification has been given; such periods were also excluded. The other labels are self-explanatory, but note that the left and right canter are included as separate gaits because it is important to demonstrate that the TS can distinguish between them.

The TS calculates the gait label every time a hoof-on event is registered from any leg. Hence, the gait labels are produced at a variable rate from about 4 Hz to 10 Hz, depending on the horse’s activity. A sliding window of 1 s length with a step of 0.5 s was used to produce a timeline for analysis. For each window, all gait labels falling within that window were collected, and the most-frequent gait label was taken and paired with the timestamp from the middle of the window. The timestamp of the first sample in the log was subtracted from all windows, which resulted in a timeline of gaits starting at 0 s and progressing forward at 0.5 s intervals for the whole session.

With the two time series acquired, the series from the TS should be regarded as the “predicted value” set, and the series aggregated from the judges is the “true value” set. For each video, an observation window was defined, where we have both a TS prediction and the ground truth labels generated by judges. We took the timestamp from the predicted value and retrieved the ground truth value at that given time to make a prediction–truth pair, or “test case”.

As mentioned above, when aggregate judge classifications resulted in −2 or −1, the test case was discarded. In addition, it was appropriate to discard test cases that were very close to transitions (as defined by the TöltSense or judges). In other words, leeway should be allowed for both the TS and judges when it comes to identifying and registering the moment of a transition. There are several reasons for this. First, there was always a delay between the horse making the transition, the judge recognising the transition has occurred, and then, again, a delay before the gait button is clicked in the web application. The TS may already have correctly recorded the transition when the judge clicks the button, so a false negative will be produced. Second, in cases of momentary loss or the change of gait (for example, a few steps of trotty tölt in the middle of a section of trot going straight back to trot), a judge is unlikely to react fast enough to register the gait change. If he/she does, it is likely to be registered late.

On the other hand, the TS is very likely to register the momentary gait change, and so, again, a false positive or false negative may be produced. There are also cases where the human eye (and hand) is quicker to register the gait change than the TS. The main example is the transition to walk, where the stride frequency is low, and it takes longer for the TS to build a buffer of steps to analyse as opposed to when going into a fast tölt, for example. In such cases, the judges may log a transition before the TS does, which results in a false negative. For these reasons, we applied a 1 s exclusion period for transitions (as identified by either the TS or the judges) as a fair degree of leeway. Any test cases falling within these exclusion periods were removed from the analysis.

### 2.3. Dataset and Labelling—Gait Glassification with a Mobile Phone

The mobile phone sensor data were collected and labelled between May and August 2021 using the TöltSense (TS, https://toltsense.com/, accessed on 1 May 2022) system (see Figure 1). The data were collected on a horse farm in southern England and across various horse farms and training centres in Iceland. Seventeen different Icelandic horses and fourteen different riders were used for the measurements, and the phone was placed in a pocket on the rider’s clothing, chosen by the rider. The phone location varied between riders with the phone placed in pockets on either trousers or jackets. The horses were ridden on different surfaces outdoors, on a track, sandy arena, or a trail. In this manner, 5.8 h of labelled data were collected, which corresponded to thousands of short segments for each gait.

The sensors from the TS system were attached to the lateral aspect of the metacarpal or metatarsal bone and set to a sampling frequency of 125 Hz, an acceleration range of ±16 g, and an angular velocity of 2000 deg/s. The TS system was responsible for synchronization between sensors and data processing, as well as capturing the phone sensor data into a log file and appending a gait label to each sample. It performs limb stride parameter calculations by detecting hoof-on/-off times using a built-in algorithm. The results of these calculations are then used to generate the gait labels. The TS system outputs 10 different labels, as can be seen in Table 2. The gait labels were the only data used from the TS system in this study, and they were generated whenever a hoof struck the ground, which was up to ten times a second for a fast tölt, i.e., four times per stride with up to 2.5 strides per second.

The phone models used for this study were different models of Samsung phones and iPhones. The data logged from the iPhones were sampled at a 50 Hz rate, whereas the data from the Samsungs were sampled at a higher rate, ranging from 100–1200 Hz, depending on the model. The data consisted of measurements from the accelerometer and gyroscope, which were subsequently rotated to a given frame of reference. One rotation was to the world-frame using a quaternion generated by the phone. We note that rotating the movement signal to the world-frame is standard practice and has been suggested by earlier studies on equine gait analysis [9,16]. Furthermore, such a rotation makes the signal more interpretable since it isolates acceleration in the vertical axis to a single dimension in the input signal. However, the world-frame does not give a clear indication of how the signal is varying in the lateral dimensions with respect to the horse. For that reason, we also studied a rotation to the horse’s frame of reference. We used the 1 Hz GPS signal to acquire the horse’s running direction in the *x*-*y* plane as an angle in the range [0,360) by comparing two consecutive longitude and latitude measurements. To determine the direction, we used a smoothed version of the horse’s direction in the *x*-*y* plane, where we averaged the degree in which it was moving over a 1 s window. To compute a circular average, we unwrapped the signal by changing elements that had an absolute difference from their predecessor of more than 180 degrees to their period-complementary value. After averaging, we wrapped the signal again to obtain a direction in the range [0,360). The direction and speed were obtained using the Android/iOS location APIs.

For the sake of clarity, we note that the model receives six dimensions as the input, acceleration around the *x*-, *y*-, and *z*-axis and angular velocity along the same axes in the given frame of reference. We also explored the variations of the input signal, by adding speed as a dimension in the input signal or only including data from the accelerometer or gyroscope.

### 2.4. Pre-Processing of Signals from Mobile Phone Sensors

The data were pre-processed using common libraries for Python: NumPy, Pandas, and PyTorch. The rides that were sampled at more than 50 Hz were downsampled to 50 Hz. It may be noted that previous results indicate that downsampling to 50 Hz does not have a large impact on gait classification performance [11] or vertical movement symmetry measures in trot [17].

We split the data into a training set and a test set to measure generalization performance. The test set contained data from four horses that were not observed in the training set and a rider/horse combination that was not in the training set (see Table 3). We split the data into segments, as is illustrated with the blue rectangle in Figure 1. When generating the segments, we selected a segment such that it overlapped with the previous segment by 90%. We chose a fixed proportional overlap between segments instead of an absolute segment shift to reduce overfitting when studying longer intervals. For this reason, we had less training and test data for longer intervals. However, since relatively little flying pace data were present, we generated segments every 20 milliseconds for the flying pace for every window size.

As in the validation of the TS, segments were discarded that were within a 2 s period of a gait switch (here, a gait switch occurs when the Töltsense labels change) to leave out periods where the horse is in transition as such transitions might not have a reliable label. This discarding further eliminated transient labelling errors that may arise due to latency in the classification from the TS.

**Figure 6:**
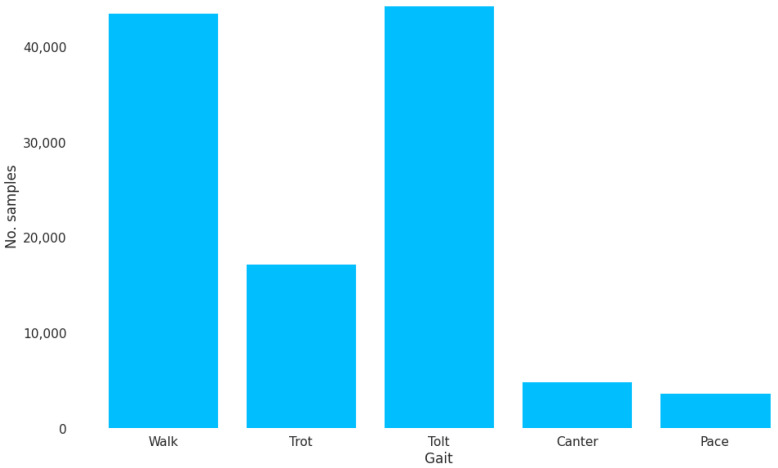
The distribution of horse gaits in the dataset for 1.5 s segments.

The ten labels from the TS system were mapped to a set of five labels representing the five gaits of the Icelandic horse (Table 2). Note that the cross canter is typically a result of rider error when transitioning to canter or flying pace (incidentally, pace horses often wear special over-reach boots to protect against strike injuries made more likely by cross canter). The two cross canters were mapped to canter, but no examples of it were actually collected in the training data. The distribution of the gaits in the training set can be seen in Figure 6.

The training set was split into a training and validation with an 85/15 split, and the samples were shuffled for training. The leave-one-outtest set contained rides from Horse Nos. 1, 2, 8, 9, and 11 while the rest were used for training. Rides from Subject No. 2 were used in both sets, but the rides had different riders. However, the rides contained a different phone model and a different rider.

### 2.5. The Gait Classification Model

The main model used for gait classification in this study was a recurrent neural network (RNN), namely, a long short-term memory (LSTM), an architecture widely used for time series classification and regression tasks [10]. Other similar RNN architectures were also tested such as a gated recurrent unit (GRU, [18]) and a bidirectional LSTM [19]. A 1-dimensional convolutional neural network was also tested.

**Figure 7:**
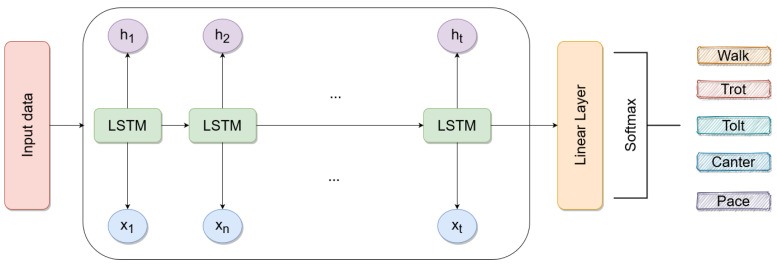
The model architecture used in this study.

The LSTM model contained a single LSTM layer with 200 units; the Bi-LSTM model had 400 units; the GRU model had 200 units. For the 1-dimensional CNN model, we had four layers with a kernel size of 3. The layer dilation rates were 12, 8, 4, and 1; the number of inputs was $n_{i}$, 16, 32, and 64, where $n_{i}$ is the number of input features used; the number of outputs was 16, 32, 64, and 128. In all models, the final layer added was a 128-dimensional linear layer (see Figure 7 for the LSTM model). The models were trained for 20 epochs with a batch size of 64 using the ADAM optimizer [20] and early stopping monitoring the loss of the validation set. All models were trained using cross-entropy as the loss function.

The model’s input was the output of the mobile sensors rotated to a given frame of reference where one axis (*z*) represents the vertical dimension and the others (*x* and *y*) represent the horizontal dimensions. In the world-frame, the horizontal axes correspond to the south–north and east–west directions. In the horse-frame, they correspond to the front–back and left–right directions. We also studied the effect of adding speed as an additional input feature, where the speed was calculated based on the GPS coordinates. The acceleration signal had three dimensions; the gyroscope signal had three dimensions; the speed signal had one dimension.

### 2.6. Smoothing Classifier Output

The model prediction only took in a segment of mobile sensor data and output a label. Due to the training approach, the model might make mistakes, which can be easily corrected on sequential data. As an example, because the model is not given its output for the last segment, it can claim that a horse is performing tölt and then brief walking for 100 milliseconds and then tölt again. Intuitively, horses do not perform such a switch from one gait to another and back within a 100-millisecond time period.

For this reason, two post-processing methods were used for smoothing the classifier output that could be applied to the leave-one-outtest datasets since they were in sequential order. The former method makes use of the linear layer of the network, which outputs a probability vector of the labels. The vector output of the softmax layer is updated with respect to the vectors preceding it in time using the exponential weight decay defined as:

(1) $$z_{0}=h_{0},\;\mathrm{and}\;\;z_{t}=\frac{z_{t-1}+h_{t}}{2}.$$

**Figure 8:**
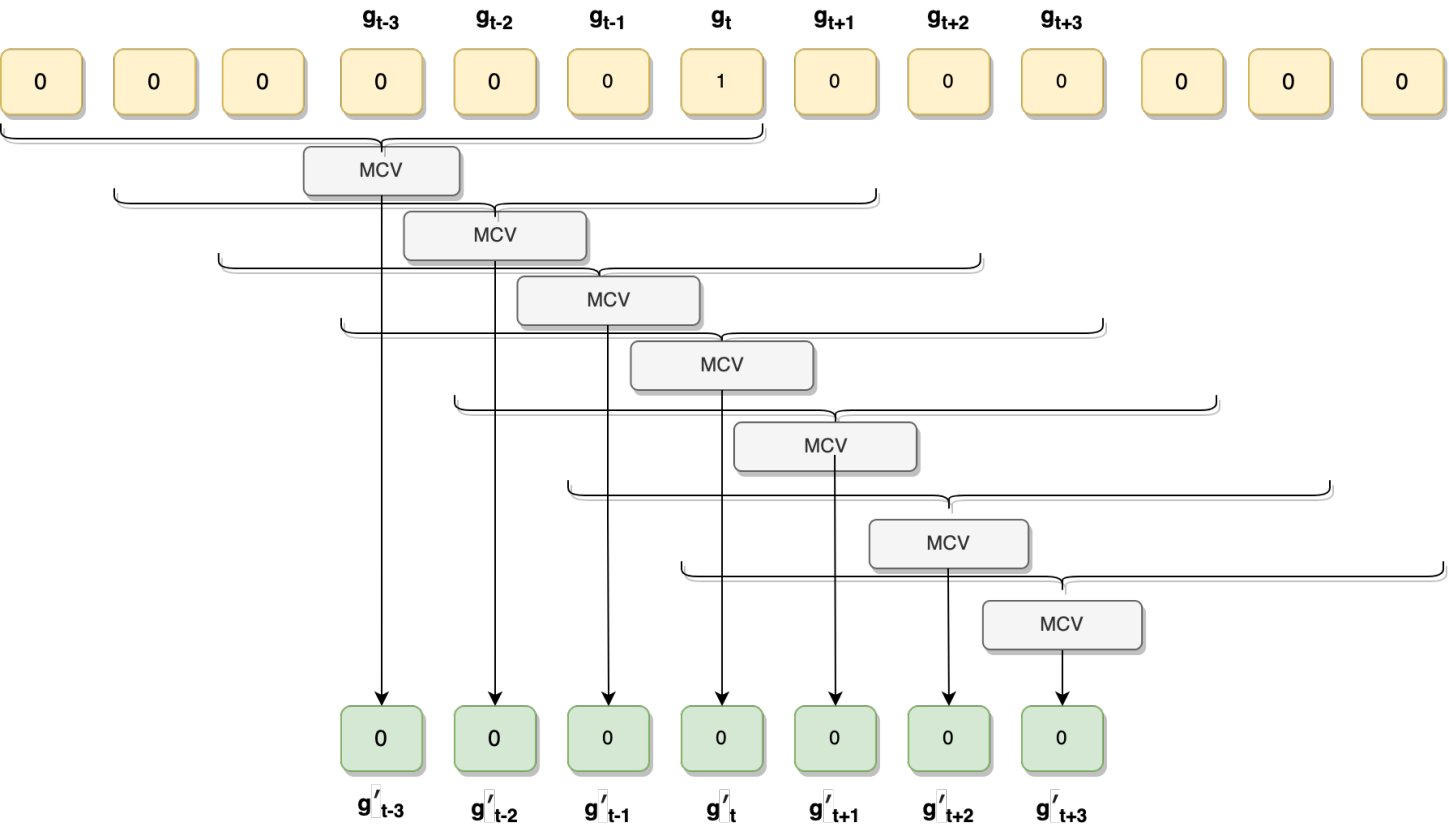
Sliding window majority vote. Here, $g_{t}$ represents the chosen gait after the first pre-processing step and $g_{t}^{\prime }$ represents the chosen gait after the majority vote. MCV is an abbreviation for “Most-Common Value”, and the curly brackets represent the window on which the most-common value is selected.

The vector h denotes the output from the LSTM and z the refined vector after using exponential decay. This method ameliorates the problem to some extent, but does not fix it completely. To further refine the result, we applied a majority vote window of size 7, which we slid over the gaits as determined by the $z_{t}$ vectors (for an illustration, see Figure 8). Concretely, we define

(2) $$g_{t}=\arg\max z_{t}$$

as the gait chosen at time *t* through the largest component of $z_{t}$. We then define $g_{t}^{\prime }$ as the most common gait in the set

$$\left\{g_{t-3},g_{t-2},g_{t-1},g_{t},g_{t+1},g_{t+2},g_{t+3}\right\}$$

where ties are broken by using $g_{t}$. The sequence $g_{t}^{\prime }$ represents the output of our method after the two post-processing steps.

By applying the post-processing step, the prediction depends not only on past predictions, but also on future predictions, smoothing the signal.

### 2.7. Performance Measures

To measure the performance of the model across all gaits, we used the micro-averaged classification accuracy defined as

(3) $$\frac{\mathrm{number}\;\mathrm{of}\;\mathrm{correctly}\;\mathrm{classified}\;\mathrm{examples}}{\mathrm{total}\;\mathrm{number}\;\mathrm{of}\;\mathrm{examples}},$$

which corresponds to the sum of the diagonal in a confusion matrix divided by the sum of all entries. For the accuracy of particular gaits, we report the one-vs.-all accuracy, i.e., where the classification is viewed as a binary classification problem with the gait in one class and all other gaits in the other class. We further report the macro-averaged gait classification accuracy where we averaged single gait classification accuracies over the gaits.

## 3. Results

### 3.1. Validation of TöltSense Labels

A total of 4421 one-second windows were generated from the TS logs, which included 179 gait transitions. Excluding cases that were subsequently marked as −2 or −1 and without exclusion periods, 4371 valid test cases were generated. Factoring in transition exclusion periods reduces the number of valid test cases (see the table below). The accuracy of the TS predictions was calculated simply as the total number of correct predictions divided by the total number of valid test cases.

In Table 4, we show how the calculated accuracy varied when we altered the exclusion period for both judge-identified and TS-identified transitions.

**Figure 9:**
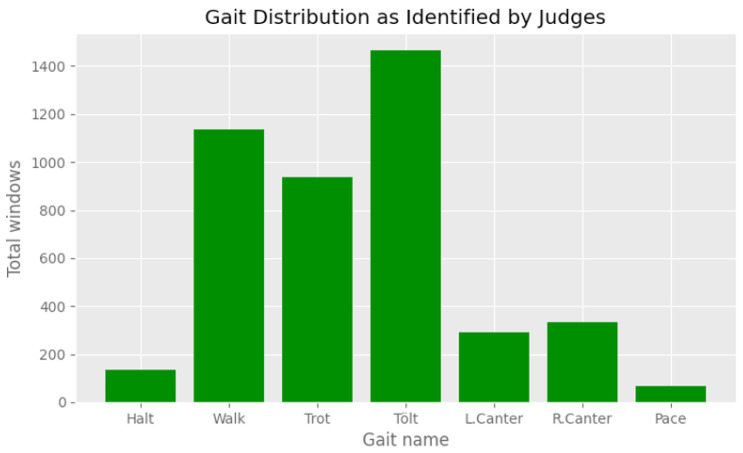
Distribution of gaits as identified by the judges. We note that pace is under-represented.

**Figure 10:**
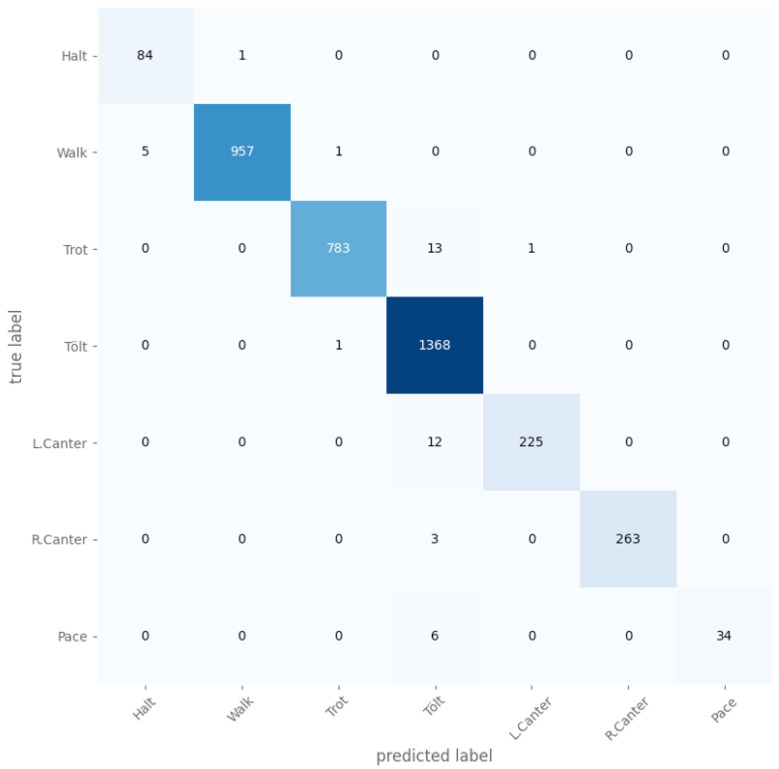
A confusion matrix for the case where both the TS and judge exclusion period was 1000 ms.

**Figure 11:**
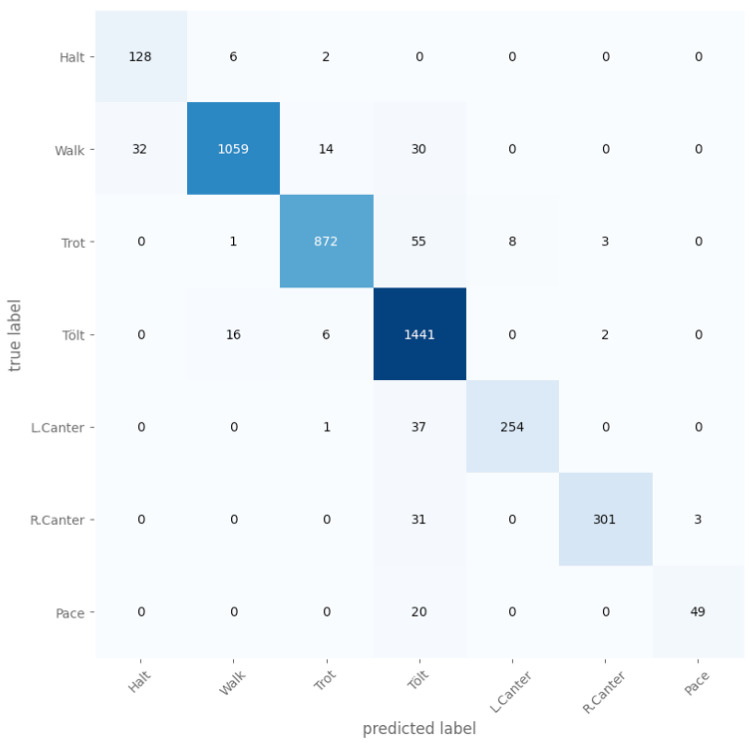
A confusion matrix for the case where both the TS and Judge exclusion period was 0 ms.

The exclusion period was varied from 0 s to 2 s, and it was found that the longer the period, the greater the accuracy was. Without any exclusion periods, the micro-averaged accuracy was 93.89%, and with 2 s for each, the micro-averaged accuracy was 99.73%. The gait distribution is shown in Figure 9, and the confusion matrix for the case where both the TS and judge exclusion period was 1000ms is shown in Figure 10. The confusion matrix without any exclusion periods is shown in Figure 11.

### 3.2. Comparing Sequence Models on Mobile Phone Sensor Data

The results of the mobile phone sensor study were based on data collected from 17 horses (see the Methods Section). When comparing sequence models, cross-validation was used where each horse was left out. For other experiments, we used a separate test set. Four of the horses were left out from the training data to measure the test performance. Since only a few horses were able to perform flying pace, we had one horse both in the training and test data, but the rider was different in each set.

We compared several models in the gait classification task using cross-validation, where each horse was left out separately. The micro-averaged classification accuracy, averaged over the horses, is shown in Table 5. All models had accuracy scores exceeding 90%, with the Bi-LSTM model reaching the highest at 94.4%.

For each horse, detailed results based on the Bi-LSTM model are shown in Table 6. We observed the highest accuracy for walking at 97%. Canter was at 94%, flying pace at 93%, tölt at 89%, and trot at 82%. However, for four horses, the micro-averaged accuracy was worse (see the Discussion Section). The model performed well, as we hypothesized. Specifically, the model did not confuse the gaits of the Icelandic horse, tölt and flying pace.

### 3.3. Using the Horse’s Frame of Reference Improves Classification Performance

Using the world-frame as a frame of reference isolates vertical variations in acceleration to a single axis. However, rotations to the horse-frame can further isolate variations in acceleration along the left–right axis and the front–back axis. We thus hypothesized that rotation to the horse-frame could lead to better classification performance. We trained an LSTM network with 200 units on 1.5 s-long segments of accelerometer and gyroscope measurements from 17 horses (see Table 3 for information about the horses). The distribution of the collected segments is shown in Figure 6. The dataset was quite imbalanced, but with the data acquisition approach, we managed to collect thousands of segments for each gait.

**Figure 12:**
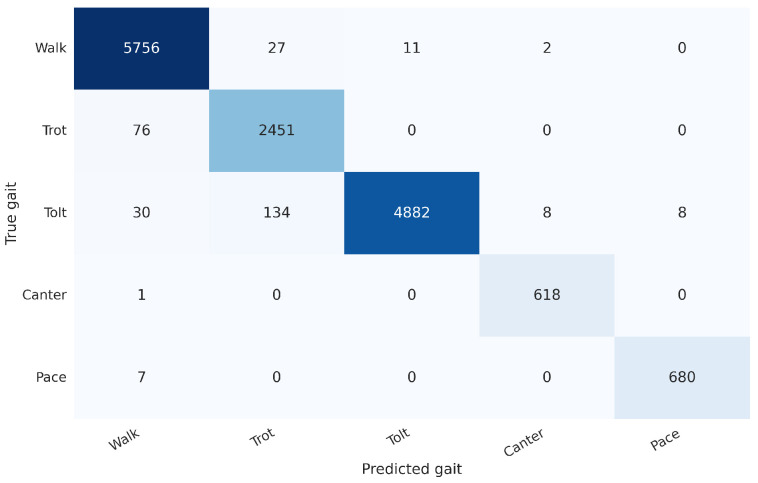
Confusion matrix for the gait classification model on the test set with 98.0% accuracy (best out of 9 random seeds with average accuracy at 96.1% and median accuracy at 95.8%). We use an input interval of length 1.5 s, where the input signal is aligned to the horse’s frame of reference.

**Figure 13:**
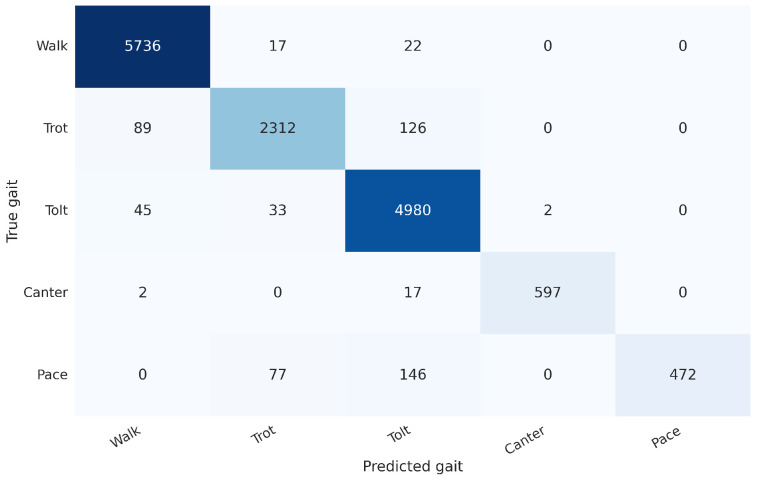
Confusion matrix for the gait classification model on the test set with 96.1% accuracy (best out of 9 random seeds with average accuracy at 93.9% and median accuracy at 92.7%). We use an input interval of length 1.5 s, where the input signal is aligned to the world-frame.

We evaluated the model on data left out from five rides, and it reached an average accuracy of 96.1% when training on the signal rotated to the horse’s frame of reference (see Figure 12 for the best run) compared to an average accuracy of 93.9% for a signal rotated to the world-frame (see Figure 13 for the best run).

### 3.4. The Model Is Robust to Choice of Interval Length

**Figure 14:**
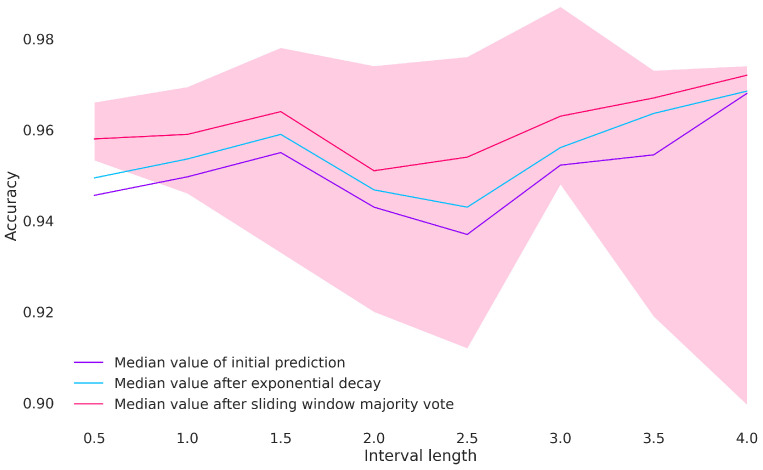
Accuracy over all gaits for each interval length (in seconds) using a rotation to the horse’s frame of reference. The blue and red lines show the result of post-processing the classification result to achieve a higher accuracy score. The shaded area is bounded by the highest and lowest reported accuracy for each interval after post-processing. The accuracy is averaged over 9 evaluations on the test set, where the model was trained using a different random seed each time.

Achieving a good performance with short intervals or less data can make predictions more responsive in an interactive environment since the model needs less time to react to gait changes. For recurrent models such as LSTMs, a shorter input can further reduce the computational cost of the inference task, which is ideal for mobile devices. For an LSTM model, the performance does not seem to depend strongly on the interval length in the range of 0.5–4 s (see Figure 14). The maximum accuracy on the test set was achieved for a 3 s interval, but the mean accuracy was highest for a 4 s interval. For an interval of length 1.5 s, the accuracy of the model averaged around 96% over nine evaluations on the test set where the model was trained each time using a different random seed. It may be noted that the variation in the test accuracy also increased with longer intervals. This can to some extent be attributed to the decreased size of the training and test sets for longer interval lengths, which leads to larger relative deviations in measures of performance on the test set.

### 3.5. Performance Comparison for Input Signal Variations

We further studied the effect of varying sampling rates and what signals we trained the model on for 1.5-s intervals when the signals had been rotated to the horse’s frame of reference. These variations reflect that not all mobile devices have a gyroscope and an accelerometer and the sampling rates vary significantly. In addition, we studied the effect that the speed had on the classification performance. The speed was derived from the GPS data on the phone’s placement and not from the accelerometer. Table 7 shows the result.

The result suggests that acceleration is the most-important input signal for gait classification. From the evaluation, we cannot conclude that the addition of gyroscope signals or speed consistently improved the model accuracy. Since acceleration reflects variations in speed, the speed signal might not be providing additional information that the model can benefit from. Furthermore, the speed signal might not be available indoors or in situations where the GPS is not reliable.

Regarding sampling rates, we did not observe a large drop in performance when they were lowered with only acceleration as the input. However, we saw a big difference in performance for 10 Hz and 15 Hz when the input from the gyroscope was used. The performance also did not clearly increase with a higher sampling rate, since we observed the best performance for 25 Hz, but not for 50 Hz.

## 4. Discussion

In this article, we presented the gait classification performance of the first phone-based classifier that recognizes all five gaits of the Icelandic horse, including flying pace. We showed that the gait classification for five-gaited horses can reach 94.4% accuracy with the model receiving only raw mobile sensor data from the accelerometer and gyroscope rotated to the horse-frame based on measurements from the magnetometer and a GPS signal. That is, specific feature engineering was not required. Instead of labelling the data by hand, we used a novel efficient approach to label the data by simultaneously collecting data from smartphones and the TS gait labelling system. That system generates a gait label based on four IMU sensors attached to a horse’s limbs, and thus, the gait label is based on a signal for the limb movements. The kind of information that the TS can generate would normally require an expert eye on the ground or an in-depth frame-by-frame analysis of video. Such circumstances inevitably incur costs and are restrictive in terms of environment, such as being confined to a riding hall, or good lighting, or fair weather. Moreover, the volume and utility of information gathered manually is limited. By contrast, the TS is a cost-effective way to collect gait labels without any external assistance or environmental restrictions.

Through cross-validation, the method achieved accuracy scores above or equal to 0.9 for all gaits except trot (0.82) and tölt (0.89). In that regard, the most-common confusion in the model was between tölt and trot. It is conceivable that more training data from different horses and riders would improve the performance on trot since the performance on the training set was better than on the test set, which can indicate mild overfitting.

Tölt is characterized by a four-beat gait in which the horse lifts its hooves off the ground in a diagonal sequence. It has half-suspension in both the front and hind. In contrast, trot is a two-beat gait in which the horse lifts its hooves off the ground in a diagonal sequence, but with a moment of suspension. Tölt is considered to be the gait between trot and flying pace. We note that tölt can be ridden at different variations, speeds, and quality [15], some of which can be hard to differentiate from trot. Experienced human observers might even disagree on trotty tölt and tölty trot, especially at higher speeds, where they might be harder to tell apart [21]. Furthermore, trot can be ridden using three main riding techniques: sitting, rising, and a two-point seat where the rider stands in the stirrups. These techniques influence how the rider moves along with the horse, and they can also influence the horse’s motion pattern [22]. We speculate that different riding techniques could make the mobile sensor movements more similar to other gaits. Together, these differences might explain the confusion in the model. To improve the performance of models relying on mobile phone sensors, it could be worthwhile to study whether different combinations of riding styles and gaits can be distinguished in mobile sensor recordings.

On the test set, the model reached a median classification accuracy of 96.9% with sampling rates down to 10 Hz. We observed a higher accuracy for 25 Hz signals than 50 Hz signals when comparing signal combinations. In principle, performance can increase by lowering the sampling rate since it translates to shorter input sequences for the model. A recurrent neural network such as an LSTM model might handle shorter inputs better [23,24]. However, lowering the sampling rate too much can cause the signal to contain less information about the underlying gait, which possibly explains the performance drop for the 15 Hz and 10 Hz signals. When exploring signal combinations, we also observed that speed did not lead to improved accuracy, in agreement with prior work [8].

To ensure the reliability of our measurements with respect to TS labels, we further validated TS accuracy in a separate study. We demonstrated a very high level of agreement between the TS and qualified sport judges when classifying the gait of Icelandic horses. The use of exclusion periods around transitions can take the agreement to over 99%, but the agreement was around 94% even without any exclusion. We earlier framed this in terms of “accuracy” by considering the judge classifications as the ground truth. In reality, we are assessing the agreement between two different measuring approaches, and it is valid to question whether the TS is more accurate than human observers in some circumstances. There are often areas where the judges disagree about the gait. For example, a very pacey tölt coming out of canter may be classed as pace by some and tölt by others. There are also cases on the boundary between walk and tölt and between tölt and trot. It may be interesting to analyse these disagreements as a project in itself. The main shortcoming of the validation study is the lack of flying pace segments.

We acknowledge that this study is limited to Icelandic horses. However, the methods we have applied to collect the data can be used to collect data for a large population of different horse breeds in diverse environments using various wearable sensors. More test data would further allow us to better measure the generalization performance. That would be especially beneficial for flying pace, the gait for which we had the fewest measurements [25]. To improve the results further, we note that it is known that sensor placement can affect model performance [9,26], and we hypothesize that phone placement could have an effect, which would explain the worse classification performance for some horse–rider pairs. We speculate that our results could be improved by making sure that factors such as the phone’s placement, phone model, riding surface, and clothing (loose vs. tight) are standardized. To mitigate the phone’s placement problem, a model could be trained that takes the placement into account, which could improve overall performance. Furthermore, to improve the user experience of an app, a model could be trained to automatically infer the phone’s placement (and possibly even the type of riding surface) such that a user would not need to assign it himself/herself. Further improvements could be achieved with better mobile phone sensors. For example, we used a 1 Hz GPS signal to estimate direction, but a higher sampling rate could allow for more detailed estimates of direction and other important parameters. For example, Pfau et al. managed to estimate essential stride parameters using a 10 Hz GPS signal [27]. Another improvement could be achieved by using several mobile sensors, such as jointly from a smartwatch and a smartphone. Estimating speed has been shown to be better with more than a single horse-mounted sensor [28], and it thus is reasonable to ask whether several human-attached sensors improve gait classification performance.

Recordings based on mobile phones open up a variety of possibilities in horse-related activity tasks. For example, it makes it feasible to study horse behaviour at a large scale through volunteer participation. Such data can be used to classify horses, define phenotypes, and possibly relate their behaviour to genomic data. Furthermore, data obtained from mobile phone sensors have been used for lameness detection [9,29], but such studies could benefit from the labelling approach we used here. Sensors attached to a horse have been used to identify lameness [30,31,32], and commercial products have been developed for that task (see, for example, https://equisense.com/ accessed on 1 May 2022 and https://equinosis.com/ accessed on 1 May 2022).

Traditionally, the human eye has been considered the gold standard for gait classification. Bragança et al. [8] claimed based on their results that human visual and subjective assessment is not optimal. Furthermore, results on lameness detection using human observers have reported low intraobserver agreement [33,34]. Therefore, when models can exceed human performance for tasks such as lameness detection, then they will possibly be better suited for data labelling. Similar to our labelling approach, data could be captured simultaneously from mobile phone sensors attached to the horse and a label from a well-performing model (we acknowledge that commercial products can be difficult to trust due to too rarely disclosed accuracies). That dataset can then be used to train a model for lameness detection. Alternatively, there is also the possibility of applying unsupervised approaches such as anomaly detection methods to detect unusual recordings at a large scale.

## 5. Conclusions

In this paper, we investigated the feasibility of using mobile phone sensors for gait classification in Icelandic horses, a breed known for its ability to perform five gaits: walk, trot, canter, tölt, and flying pace. We used the TöltSense (TS) system to label our training data and evaluated the accuracy of different machine learning models on these data. Our results showed that it is possible to achieve high accuracy in gait classification using mobile phone sensors, with the best-performing model reaching an accuracy of 94.4%.

These findings have significant implications for the field of gait classification. Mobile phones are widely available and portable devices that are often carried by individuals, making them a convenient and accessible option for gait classification. Our results suggest that mobile phone sensors can be used as a reliable alternative to sensors attached to the animal, offering a more practical and convenient solution for gait classification in certain contexts.

However, it is important to note that the accuracy of gait classification can be influenced by various factors, such as the environment in which the data are collected and the quality of the sensors. Further research is needed to fully understand the limitations and potential of mobile phone sensors for gait classification in different contexts and with different types of animals.

In conclusion, our study demonstrated the feasibility of using mobile phone sensors for gait classification in Icelandic horses, offering a convenient and accessible alternative to traditional methods. These findings have the potential to broaden the scope of gait classification research and to facilitate the development of mobile-phone-based applications for gait classification.

## Figures and Tables

**Table 1:** Gait labels used by judges. The table shows the gait labels from the TöltSense system that the judges used to label the video segments.

TöltSense Label	Number
No majority/disputed	−2
Not classified	−1
Halt	0
Walk	1
Trot	2
Tölt	3
Left Canter	4
Right Canter	5
Pace	6

**Table 2:** Mapping from the TöltSense labels to the ones used in this study.

TöltSense Label	Label
Standing	Not Used
Walk	Walk
Trot	Trot
Tölt	Tölt
L Canter	Canter
R Canter	Canter
L Cross Canter	Canter
R Cross Canter	Canter
Flying Pace	Flying Pace

**Table 3:** Overview of the horses used for the study. The total time of labelled data sums up to 5.4 h. Note that only the horses in the 2nd, 10th, and 11th row are five-gaited.

Horse No.	Location	Rides	Walk	Trot	Tölt	Canter	Flying Pace	Total Time
1	England	1	1026 s	212 s	376 s	190 s	0 s	30 min
2	England	2	1488 s	98 s	253 s	39 s	85 s	33 min
3	England	2	767 s	427 s	973 s	154 s	0 s	39 min
4	Iceland	3	470 s	416 s	935 s	42 s	0 s	31 min
5	Iceland	2	394 s	121 s	710 s	0 s	0 s	20 min
6	Iceland	4	1394 s	628 s	2275 s	88 s	0 s	73 min
7	Iceland	2	1319 s	712 s	1161 s	257 s	0 s	57 min
8	Iceland	1	354 s	192 s	496 s	63 s	0 s	18 min
9	Iceland	1	157 s	120 s	212 s	32 s	0 s	8 min
10	Iceland	2	0 s	0 s	0 s	0 s	28 s	0.5 min
11	Iceland	1	0 s	0 s	0 s	0 s	27 s	0.5 min
12	England	1	155 s	55 s	67 s	40 s	0 s	5 min
13	England	1	144 s	0 s	338 s	0 s	0 s	8 min
14	England	1	141 s	90 s	144 s	102 s	0 s	8 min
15	England	1	124 s	61 s	100 s	14 s	0 s	5 min
16	England	1	117 s	41 s	80 s	24 s	0 s	4 min
17	England	1	134 s	80 s	134 s	38 s	0 s	6 min

**Table 4:** Gait classification accuracy for different sizes of exclusion windows.

TS Excl (ms)	Judge Excl (ms)	Test Cases	Accuracy
2000	2000	3358	99.73%
1000	1000	3757	98.86%
1000	0	3909	97.95%
0	1000	3990	96.62%
0	0	4371	93.89%

**Table 5:** The average micro-averaged gait classification accuracy was computed for each horse using cross-validation, where the horse was left out for evaluation. The cross-validation was repeated five times for each model, and the results were averaged.

Model	Macro avg.
Bi-LSTM	94.4
GRU	91.2
LSTM	93.3
1D CNN	93.9

**Table 6:** We performed cross-validation where each horse was left out. For each horse, we ran the evaluation with five different initializations of the Bi-LSTM model and report the micro average for each gait. The macro average over all the gaits is 0.91. The amount of training data for each horse can be found in Table 3.

Horse ID.	Walk	Trot	Tölt	Canter	Flying Pace	Micro avg.
1	1.0	1.0	1.0	1.0	-	1.0
2	0.98	0.72	0.82	1.0	0.87	0.95
3	1.0	0.97	0.99	1.0	-	0.99
4	0.83	0.82	0.96	0.61	-	0.89
5	0.99	0.68	0.94	-	-	0.94
6	0.99	0.97	0.99	0.69	-	0.98
7	1.0	0.88	0.92	0.99	-	0.95
8	0.95	0.92	1.0	1.0	-	0.97
9	0.93	0.88	1.0	1.0	-	0.96
10	-	-	-	-	0.95	0.95
11	-	-	-	-	0.9	0.9
12	0.98	0.44	0.74	0.96	-	0.87
13	0.97	0.0	0.98	-	-	0.97
14	1.0	1.0	1.0	1.0	-	1.0
15	1.0	0.57	0.61	1.0	-	0.82
16	0.97	0.66	0.5	1.0	-	0.74
17	1.0	0.95	0.93	0.99	-	0.94
Macro avg.	0.97	0.82	0.89	0.94	0.93	0.94

**Table 7:** Classification accuracy on the test set for different signal combinations and rates for 1.5 s segments and a rotation to the horse-frame with the best performance for each rate in bold. The numbers reported are the median micro-averaged accuracy over 9 random seeds (a different random seed was used each time the model was trained and evaluated on the test set). **G** stands for Gyroscope only, **A** for Accelerometer only and **G+A** for Gyroscope and Accelerometer.

	without Speed			with Speed		
Rate	G	A	G+A	G	A	G+A
10 Hz	80.8	**96.9**	92.2	76.7	95.7	91.1
15 Hz	91.4	**97.4**	96.8	92.4	96.1	96.0
25 Hz	92.3	**97.8**	97.1	92.5	97.1	96.6
50 Hz	92.6	96.8	96.4	90.7	**97.3**	95.8

## Data Availability

The datasets used and analysed during the current study are available from Horseday ehf. upon reasonable request.

## Abbreviations

The following abbreviations are used in this manuscript:

TS	TöltSense
RNN	Recurrent neural network
LSTM	Long short-term memory
GPS	Global positioning system
GRU	Gated recurrent unit
CNN	Convolutional neural network
